# Fasting activates macroautophagy in neurons of Alzheimer’s disease mouse model but is insufficient to degrade amyloid-beta

**DOI:** 10.1038/srep12115

**Published:** 2015-07-14

**Authors:** Xigui Chen, Kanoh Kondo, Kazumi Motoki, Hidenori Homma, Hitoshi Okazawa

**Affiliations:** 1 Department of Neuropathology, Medical Research Institute, Tokyo Medical and Dental University, 1-5-45 Yushima, Bunkyo-ku, Tokyo 113-8510, Japan; 2 Center for Brain Integration Research, Tokyo Medical and Dental University, 1-5-45 Yushima, Bunkyo-ku, Tokyo 113-8510, Japan

## Abstract

We developed a new technique to observe macroautophagy in the brain *in vivo*, and examined whether fasting induced macroautophagy in neurons and how the induction was different between Alzheimer’s disease (AD) model and control mice. Lentivirus for EGFP-LC3 injected into the brain successfully visualized autophagosome in living neurons by two-photon microscopy. The time-lapse imaging revealed that fasting increased the number, size and signal intensity of autophagosome in neurons. In AD model mice, these parameters of autophagosome were higher at the basal levels before starvation, and increased more rapidly by fasting than in control mice. However, metabolism of exogenous labeled Aβ evaluated by the new technique suggested that the activated macroautophagy was insufficient to degrade the intracellular Aβ increased by enhanced uptake from extracellular space after fasting. Ordinary immunohistochemistry also revealed that fasting increased intracellular accumulation of endogenous Aβ, triggered cell dysfunction but did not mostly decrease extracellular Aβ accumulation. Moreover, we unexpectedly discovered a circadian rhythm of basal level of macroautophagy. These results revealed new aspects of neuronal autophagy in normal/AD states and indicated usefulness of our method for evaluating autophagy functions *in vivo*.

Autophagy, especially macroautophagy mediated by autophagosome, has been implicated in various neurodegenerative diseases including AD. Ultrastructural analysis of postmortem human AD brains revealed increased autophagosomes in dystrophic neurites^1^. Macroautophagy was also suggested to be a pathway of generating amyloid beta (Aβ) in the cytoplasm^2^. Meanwhile autophagy-related genes were induced in autopsy brains of AD patients^3^ and autophagosomes were co-localized not only with Aβ in AD but also with a-synuclein and tau aggregation in autopsy brains of Parkinson’s disease and frontotemporal lobar degeneration^4^, suggesting that misfolded disease proteins might generally induce autophagy.

Some neurodegenerative diseases have more direct relationships to autophagy^5^. Familial Parkinson’s disease causative proteins, PARK2/Parkin and PARK6/PINK1 act as indicators of functionally abnormal mitochondria to induce mitophagy^6^,^7^,^8^,^9^. Hereditary spastic paraparesis type 15 are linked to mutations of SPG15 gene that promotes autophagosome maturation^10^. Mutations of an adaptor protein for selective autophagy, p62 are associated with amyotrophic lateral sclerosis (ALS) 11.

*In vivo* analysis of autophagy after nutritional starvation was performed in a pioneering work by Mizushima, Ohsumi and their colleagues with LC3-GFP transgenic mice^12^, but induction of macroautophagy was not detected in the brain tissues after fixation. Meanwhile, it was reported thereafter that inhibition of mTOR induced autophagy and ameliorated polyglutamine disease pathology^13^. Moreover, autophagic response of neurons might be conditional^14^. In contrast to inducible autophagy, constitutive autophagy is established to protect neurons *in vivo* from neurodegeneration through clearance of ubiquitinated proteins^15^,^16^. The discrepancy awaits further analysis with a new technique to observe living neurons to settle down the issue of *in vivo*.

## Results

### A new *in vivo* imaging of macroautophagy in the brain of living animals based on two-photon microscopy

**Figure 1:**
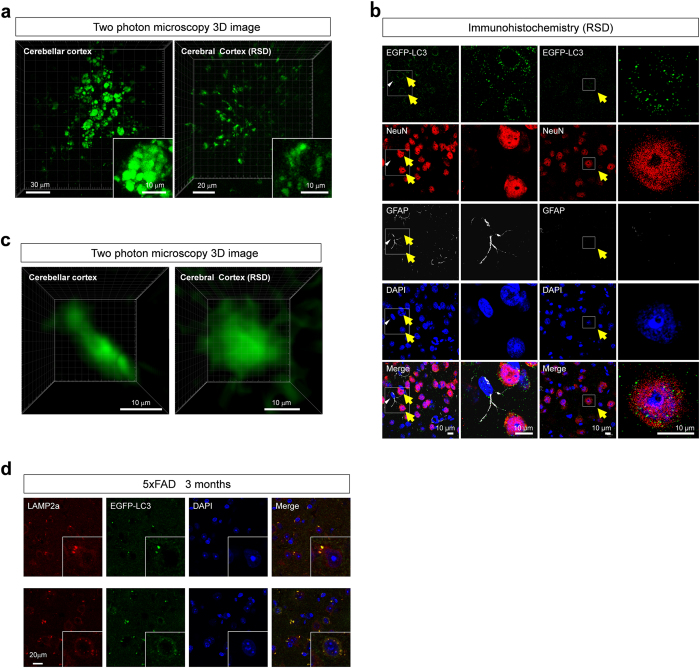
*In vivo* imaging of macroautophagy in neurons (**a**) EGFP-LC3 lentivirus was infected to cerebellar cortex (left panel) and retrosplenial dysgranular cortex (RSD, right panel) of wild type mice (C57BL/6 x SJL) at 3 months of age. Twenty days later, EGFP-LC3 signals were directly observed by two-photon microscopy. The EGFP-positive vesicles were distributed in a group as if they were autophagosomes in a cell. (**b**) The brain tissues of wild type mice injected with EGFP-LC3 lentivirus were immunostained with anti-NeuN and GFAP antibodies. EGFP-LC3 vesicles surrounded NeuN-positive neuronal nuclei (yellow arrows), indicating that they were autophagosomes in neurons. Such distributions of EGFP-LC3 were not found in GFAP-positive astrocytes (white arrowhead), suggesting that most EGFP-LC3 vesicles were located in neurons. EGFP-LC3 was directly observed without immunostaining in these experiments. (**c**) Two-photon microscopic observaion of RSD region of AAV-EGFP-injected wild type and 5xFAD mice at 3 months. Both genotypes of mice showed homogeneous signals of EGFP in cells, supporting the specificity of EGFP-LC3 signals. (**d**) Colocalization of a part of EGFP-LC3 vesicles with LAMP2A in RSD region of 5xFAD mice at 3 months.

To visualize autophagic vacuoles in living neurons in the brain, we generated lentiviral vector expressing EGFP-LC3. We injected 5 μl of lentiviral vector (titer 5.0 × 10^6^ vector genomes/ml) into retrosplenial dysgranular cortex (RSD) or cerebellar cortex. Twenty days after injection the mice were investigated by two-photon microscopy (FV1000MPE2, Olympus, Japan) with the thin skull method as described in Methods. In both areas, clustered EGFP-positive vesicles and dispersed fine EGFP-positive dots were observed (Fig. 1a). Especially in the cerebellar cortex, the vesicles with high intensities were clustered in a narrow area of 10–20 μm-diameter, suggesting that they correspond to the cell body of Purkinje cells aligned in a single layer (Fig. 1a, Supplementary Figure 1). In reconstructed images of the cerebellum, EGFP-LC3 vesicles were also aligned in the main dendrite of Purkinje cells (Supplementary Figure 1).

To verify that such clusters of EGFP-LC3 vesicles actually corresponded to the cell body of Purkinje cells, we used double transgenic mice (loxP-flanked STOP cassette Td Tomato x Ptf1a-promoter-Cre) that express red fluorescent protein (The Jackson Laboratory, B6.Cg-Gt (ROSA) 26Sortm14 (CAG-tdTomato) Hze/J, 007914) in GABAergic Purkinje cells in the cerebellum^17^. Infected lentiviral vector actually expressed EGFP-LC3 protein in Purkinje cells, a part of granule cells, but not in TdTomato-positive GABAergic neurons in molecular cell layer (Supplementary Figure 2). To verify the expression of EGFP-LC3 in cortical neurons, we performed immunohistochemistry with anti-NeuN or GFAP antibody and examined co-localization of non-stained native EGFP-LC3 with a cell-specific marker (Fig. 1b). The result revealed EGFP-LC3 vesicles/dots were distributed in NeuN-positive neurons (Fig. 1b). GFAP-positive astrocytes might also possess EGFP-LC3-positive dots, while the signals were weak in comparison to neuronal EGFP-LC3 vesicles (Fig. 1b).

On the other hand, infection of AAV-EGFP generated diffuse intracellular signals of EGFP (Fig. 1c) supporting that the EGFP-LC3 vesicles were not the artificial self-aggregates of EGFP as reported^18^. Moreover, we found by confocal microscopy that a part of the EGFP-LC3 vesicles was co-stained with a lysosome marker, LAMP2A in brain tissues, indicating that these EGFP-LC3 vesicles were actually fused with lysosomes (Fig. 1d).

### Starvation-dependent induction and circadian rhythm of macroautophagy in neurons

**Figure 2:**
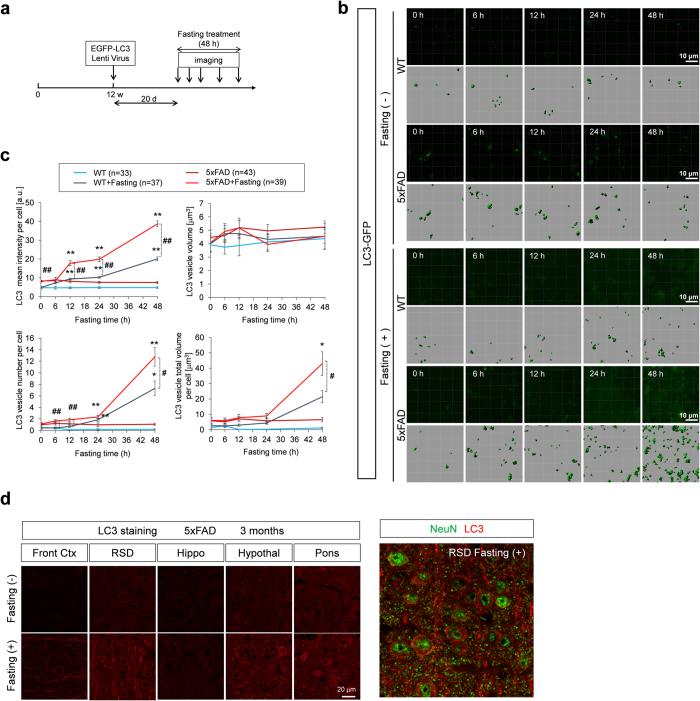
*In vivo* imaging of autophagosome in AD model and control mice (**a**) Experimental protocol of the *in vivo* time-lapse imaging to test the effect of fasting on autophagosome formation. (**b**) Time-lapse imaging of EGFP-LC3 vesicles was performed at a similar region of AD model and control mice at 3 months with or without fasting. The signal intensities were higher in 5xFAD mice than control mice before fasting. Fasting induced the increase of signal intensities in both genotypes, while the induction was more prominent in 5xFAD mice. (**c**) Quantitative analyses of the chronological changes of EGFP-LC3 vesicles with or without fasting. In mean signal intensity per cell, mean vesicle number per cell, and mean vesicle volume per cell, the values were higher in 5xFAD mice than control mice. Some of these values were also increased more remarkably in 5xFAD mice than control mice. Mean volume of the vesicles was not changed so remarkably. Mean +/− SE are shown. # or ## indicates significant differences between WT+Fasting and 5xFAD+Fasting groups. * or ** indicates significant differences between fasting (+) and fasting (−) groups in the same genotype. #p < 0.05; ##p < 0.01; *p < 0.05; **p < 0.01 in Two-way ANOVA followed by post hoc Tukey’s test. (**d**) LC3 staining of various brain regions revealed fasting treatment generally increased macroautophagosomes.

Since these results supported usefulness of two-photon microscopic observation of EGFP-LC3 for evaluation of macroautophagy, we applied the technique to answer the questions whether fasting treatment induces macroautophagy in neurons and how the autophagic response is different between 5xFAD mice, one of the severest mouse AD models that firstly shows Aβ deposition at 3 months of age, and the background mice, C57BL/6 x SJL (Fig. 2). We firstly examined the effect of fasting treatment on body weight and blood glucose, and confirmed that 5xFAD and background mice showed similar responses to fasting in these parameters (Supplementary Figure 3). In this experiment, 20 days after injection of EGFP-LC3 lentivirus, the mice were fasted and supplied only with water for 48 hours (Fig. 2a). Two-photon microscopic observation was performed at 0, 6, 12, 24 and 48 hours time points during fasting (Fig. 2a). Using the vessels as markers, the position of observation was strictly controlled (Supplementary Figure 4).

Before analyzing the effect of fasting, we needed to test whether autophagosome formation possesses a circadian rhythm (Supplementary Figure 5) because it had not been investigated previously. Unexpectedly, our live imaging of the brain revealed that the number, volume, and signal intensity per cell of the EGFP-LC3 vesicles changed in a circadian rhythm pattern (Supplementary Figure 5). All the parameters increased during daytime (light) and decreased in nighttime (dark). Interestingly, however, the parameters started to decrease around 4 PM when mice do not eat much (Supplementary Figure 5), suggesting that the circadian rhythm genes might affect autophagosome formation independently of feeding behavior. However, the question whether circadian rhythm genes affect autophagosome formation through or not through feeding behavior is an open question requiring further investigation.

Therefore we started the observation strictly at the same time points to evaluate the response of autophagosome to the fasting treatment (Fig. 2a, b). Two photon microscopy images (100 μm x 100 μm x 100 μm volume) were obtained from four groups of mice. The number, signal intensity and volume of EGFP-LC3 vesicles were quantified and their mean and SD were calculated (Fig. 2c). More than five mice were analyzed in each group of 5xFAD fasting, 5xFAD non-fasting, Wt fasting and Wt non-fasting mice, respectively (Fig. 2c). Detailed methods for acquiring these parameters were described in Methods. The time-lapse live imaging revealed that basal levels of EGFP-LC3 vesicles were higher in 5xFAD mice at the number, intensity, and total volume of vesicles per cell but not the average size of puncta (Fig. 2c). In addition, when these values were corrected by the basal values in each mouse group, the increasing ratio was also higher in 5xFAD mice (Fig. 2c).

We also employed an ordinary immunohistochemsitry method with postmortem brains of 5xFAD mice after fasting treatment to detect endogenous LC3 vesicles. In this analysis, sensitivity of the endogenous LC3 detection was far lower than that of AAV-EGFP-LC3 by our new method, and it was hard to evaluate the number of macroautophagosome strictly. However, the result of LC3 signals still suggested induction of macroautophagosome after fasting, which was generally observed across multiple brain regions (Fig. 2d).

### Effect of starvation-induced macroautophagy on extracellular and intracellular Aβ accumulation

**Figure 3:**
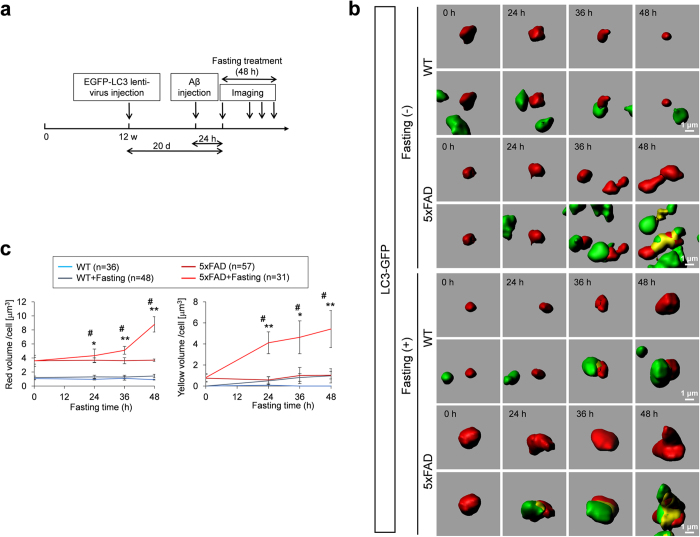
Endocytosis and autophagosome-dependent degradation of Aβ by cortical neurons (**a**) Experimental protocol of the chronological *in vivo* imaging to test Aβ uptake and degradation in the cortical neurons is shown. (**b**) Time lapse imaging of TAMRA-β-amyloid (upper panels) and TAMRA-β-amyloid + EGFP-LC3 vesicles (lower panels) at 24(0), 48(24), 60(36) and 72(48) hours after injection of TAMRA-β-amyloid. Interaction between endosomes (red) and autophagosomes (green) were observed chronologically. (**c**) Quantitative analyses of the chronological changes of endosomes (TAMRA-β-amyloid-positive vesicles, left graph) and secondary lysosomes (TAMRA-β-amyloid and EGFP-LC3 double positive vesicles, right graph) with or without fasting. Volume per cell was calculated. # or ## indicates significant differences between WT+Fasting and 5xFAD+Fasting groups. * or ** indicates significant differences between fasting (+) and fasting (−) groups in the same genotype. #p < 0.05; ##p < 0.01; *p < 0.05; **p < 0.01 in Two-way ANOVA followed by post hoc Tukey’s test. (d) Staining with anti-LC3 antibody of 5xFAD mice with or without fasting revealed increased autophagy at various brain regions.

Finally, we tested whether induced autophagosome was really effective for degradation of Aβ, because it was reported previously that AD-asscociated mutation of presenilin-1 impairs autolysosome acidification and cathepsin activation to inhibit proceeding of autophagy processes^19^. Such a dysfunction in macroautophagy might occur in 5xFAD mice and might prevent degradation of the substrates within autophagosomes after fusion with lysosomes. For this purpose, we injected Aβ labelled with TAMRA into the retrosplenial dysgranular cortex (RSD), the brain area that corresponds to human precuneus, and observed dynamics of Aβ by time-lapse imaging for 2 days from 24 hours after injection (Fig. 3a). The experiment might mimic over-secretion of Aβ from hyperactivated neurons in brain regions composing the default mode neural network.

First we found that injected Aβ was taken up into neurons within 24 hours after injection (Fig. 3b). Importantly, the amount of Aβ (red vesicle) in neurons was obviously higher in 5xFAD mice (Fig. 3b,c). The increase of intracellular Aβ could be explained by increased uptake of extracellular Aβ by endocytosis. Analysis of the yellow vesicle volume per cell that reflects secondary lysosome, the fused vesicle of endosome and autophagosome^20^, revealed that the amount of secondary lysosome increased in neurons during the time of fasting whereas in which Aβ remained undegraded (Fig. 3c). The increase of such “residual body”^20^ containing Aβ was observed both in wild type and 5xFAD mice, while the extent of increase was more remarkable in 5xFAD mice (Fig. 3c).

**Figure 4:**
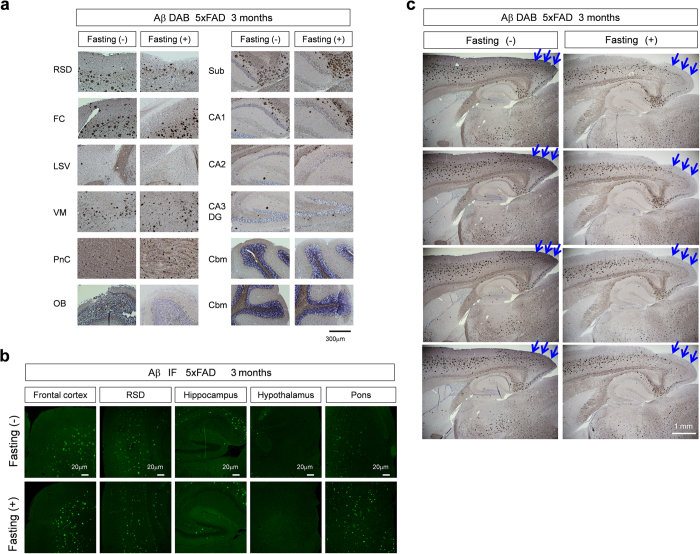
Fasting treatment does not affect extracellular Aβ accumulation(**a**) Images of Aβ at various brain regions of 5xFAD mice with or without fasting at 3 months. DAB satin was used to visualize the antibody reaction. No obvious difference was detected in intracellular and extracellular Aβ accumulation between fasting (−) and fasting (+) groups at all brain regions. RSD, retrosplenial dysgranular cortex; FC, frontal cortex; LSV, ventral part of lateral septal nucleus; VM, ventromedial thalamic nucleus; PnC, caudal pontine reticular nucleus; OB, olfactory bulb; Sub, subiculum; CA1, hippocampus CA1; CA2, hippocampus CA2; CA3, hippocampus CA3; DG, dentate gyrus; Cbm, cerebellum (**b**) Images of Aβ visulaized by fluorescent secondary antibody at various brain regions of 5xFAD mice with or without fasting at 3 months. No obvious difference was detected in intracellular and extracellular Aβ accumulation between fasting (−) and fasting (+) groups at all brain regions. (**c**) DAB staining of sagittal sections revealed that extracellular Aβ accumulation was decreased in visual cortex (arrows).

For the comparison of the effect of fasting treatment on Aβ accumulation across different regions of the brain, we employed a method using the post-fixed brain samples of 5xFAD mice at 3 months taking the advantage of the pathological stage. In this case we observed the effect of fasting on accumulation of endogenous Aβ of 5xFAD mice instead of exogenous Aβ-TAMRA. The DAB and fluorescence immunostainings with anti-Aβ antibody revealed no significant difference of extracellular Aβ accumulation between non-fasting and fasting groups of 5xFAD mice in most brain regions (Fig. 4a,b). However, we detected a tendency that extracellular Aβ accumulation was decreased in visual cortex in fasting groups of 5xFAD mice (Fig. 4c).

**Figure 5:**
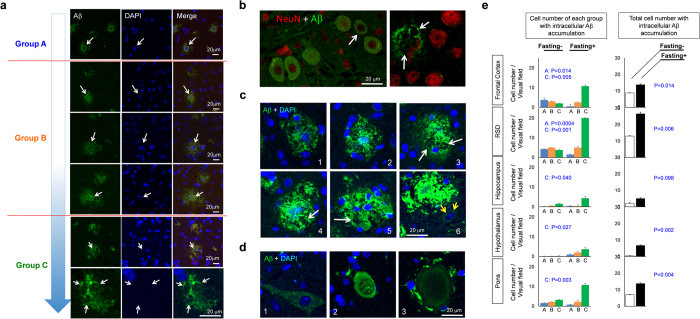
Intracellular Aβ accumulation was affected by fasting(**a**) Various forms of intracellular Aβ accumulation observed in the cortex of 5xFAD mice at 3 months. Accumulation of intracellular Aβ often accompanied with blurring or fading out of the nuclei, thus they were aligned with hypothetical progression of the cell pathology. Intracellular Aβ -positive cells with intact nuclei, with abnormal nuclei, and with no nuclei are classified to group A, B and C, respectively. (**b**) Co-staining of NeuN and Aβ revealed that neurons are the cells possessing intracellular Aβ accumulation. (**c**) Hypothetical progression of extracellular Aβ aggregates from the seed of intracellular Aβ accumulation is shown with representative images in RSD of 5xFAD mice at 3 months. White arrows suggest the defect of dead neurons. Yellow arrows suggested a enlarged nucleus with abnormal DAPI stains of the nucleus. (**d**) Abnormal ballooning of cells with intracellular Aβ accumulation suggesting atypical cell death. (**e**) Quantitative analysis of the number of group A, B and C cells at various brain regions in fasting and non-fasting 5xFAD mice at 3 months. P-values for comparison in each group or total cell numbers between fasting and non-fasting were calculated by Welch’s test. N = 4.

Intracellular Aβ accumulation was also confirmed across different brain regions as we reported previously^21^. Also unexpectedly, we observed that intracellular Aβ accumulation accompanies blurring or fading-out of DAPI or NeuN stains of the nuclei of neurons (Fig. 5a,b) suggesting that a certain type of cell death was induced by intracellular Aβ accumulation and that extracellular plaque formation might be triggered by the seed of Aβ foci of ghost neurons (the residual intracellular Aβ accumulation of dead neurons). Indeed, we often observed various images supporting the progression from intracellular to extracellular Aβ aggregates (Fig. 5c), and queer balloonings of the cytoplasm and apoptoic changes of the nuclei in intracellular Aβ positive cells (Fig. 5d).

Hence, we categorized such types of cells with intracellular Aβ accumulation into three groups, vital cells with clear nuclear margin with DAPI stains (Group A); dying cells with blurred or faint DAPI nuclear stains (Group B); and dead cells with the defect of DAPI nuclear shape (Group C) (Fig. 5a). Quantitative analysis revealed that the total cell number of neurons with intracellular Aβ accumulation was increased by fasting treatment generally in all brain regions (Fig. 5e). Quantification of each group revealed that fasting treatment induced a shift from A to C generally in all regions of the brain (Fig. 5e).

These results were consistent with the hypothesis that intracellular Aβ accumulation triggers the cell death and that plaque formation is seeded by the ghost of intracellular Aβ accumulation, whereas this hypothesis should be examined more extensively by employing additional methods in the future.

## Discussion

Our study firstly proved that macroautophagy was actually induced by starvation in mouse neurons *in vivo*. This finding was consistent with a previous result with post-mortem analysis of GFP-LC3 transgenic mice after fasting^22^. The GFP-LC3 transgenic mice were starved for 24 or 48 hours, perfused and fixed. After killing the mice, the authors of the previous study sampled the brain tissues and observed the GFP-LC3 fluorescence. Compared with their method, our technique has an advantage to directly observe the change of macroautophagy in the brain of living animals but not of dead animals. On the other hand, the protocol used in this study was limited to observation at one or several restricted regions of the brain. However, since our AAV vector is also applicable for systemic delivery by intravenous injection, it might be possible to overcome the limitation of our method in the future.

The finding in this study that fasting induces macroautophagy basically supports previous results showing that activation of macroautophagy by chemicals or vectors ameliorated the pathology of neurodegenerative diseases in various animal models. In addition we unexpectedly discovered circadian rhythm of neuronal macroautophagy *in vivo*. The circadian rhythm might be interesting if we consider it with the previous finding that Aβ secretion is reduced during the sleep^23^. These results would collectively contribute to understanding of the significance of autophagy in the brain.

Our results also suggested even though autophagy was activated in 5xFAD mice under starvation, the intracellular degradation of Aβ was still insufficient to compensate the increased uptake of Aβ from extracellular space (Fig. 3). The idea was further supported by immunohistochemistry of 5xFAD mice showing that fasting treatment enhanced intracellular accumulation of endogenous Aβ (Fig. 5). Moreover, fasting did not largely affect extracellular accumulation of endogenous Aβ in 5xFAD mice (Fig. 4a,b). If this is the case in human AD pathology, such enhanced uptake of Aβ by calorie restriction could be harmful for neuronal function if the intracellular Aβ triggers abnormal signaling cascade. Supporting this hypothesis, we observed increased number of neurons with intracellular Aβ that lost viability after fasting treatment (Fig. 5).

However, it is also of note that we had injected a high amount/concentration of Aβ to visualize the metabolism *in vivo* and that expression level of Aβ in 5xFAD mice was extremely higher than in human AD patients. Therefore, starvation–activated macroautophagy might still ameliorate the Aβ pathology if Aβ concentration in extracellular space is not so high. In that case, induction of Aβ uptake by starvation might reduce the extracellular Aβ and the up-taken intracellular Aβ at a relatively low level could be degraded by activated macroautophagy. Then, the resultant decrease of extracellular Aβ might also rescue the synaptic transmission. The critical point of concentration of the two hypotheses needs further investigation.

Collectively, the balance among secretion, endocytosis and degradation of Aβ should play a pivotal role in initiation and progression of human AD pathology. Therefore nutritional condition and circadian rhythm, which may be influenced by our life style, are considered to be intriguing factors for AD.

In conclusion, this study revealed that nutritional starvation induces macroautophagy in neurons but the induction is insufficient to degrade a high amount of Aβ in AD-associated pathological condition.

## Methods

### AD model mice

5xFAD mice and control mice (male, 3 month old) were used for the experiments in this study. 5xFAD transgenic mice express mutant human APP770 with triple mutations: Swedish (KM670/671NL), Florida (I716V), and London (V717I), and mutant human PS1 with double mutations: M146L and L285V under the control of mouse *Thy1* promoter^24^. The background was C57BL/6 x SJL. Mice were kept under standard laboratory conditions: 12 h light and 12 h dark; light on at 8:00 am, off at 8:00 pm at 22 ± 1 ^o^C and water and food were supplied ad libitum.

### Generation of lentiviral vectors

For construction of pEGFP-LC3/pLVSIN-CAG, Nhel-BamHI fragment digested from pEGFP-LC3^25^ was subcloned into pLVSIN-CMV and CMV promoter was replaced by CAG promoter with Clal-XhoI. The pEGFP-LC3/pLVSIN-CAG was cloned into transfer vectors. Using Lenti-X^TM^ Expression Systems (Clontech Laboratories, Inc), 6 μg of pEGFP-LC3/pLVSIN-CAG in Lenti-X HTX packaging Mix (components of Lenti-X HTX packaging Mix) was transfected into 293T cells (plated at 4 × 10^6^ cells 16 hours before) with Lipofectamine^®^ 2000 (Life Technologies; Cat.11668–019) following the manufacturer’s instruction. 48 hours after transfection, medium of the transfected cells was collected and centrifugation with 8,000 *g* for 5 min at 4 ^o^C. The supernatant was ultra-centrifuged with 48,000 *g* for 2 hours at 4 ^o^C). The pellet was suspended in PBS and centrifuged again with 8,000*g* for overnight at 4 ^o^C. The pellet was suspended with 160 μl of PBS by pipetting 100 times, kept at 4 ^o^C for 1hour, pipetted 100 times, kept at 4 ^o^C for 1hour, centrifuged with 8,000 *g* for 5 min at 4 ^o^C, and the final supernatant was kept at −80 ^o^C before experiment.

### Titration of viral vectors

Viral genomes were quantified using a qPCR cycler (ABI 7500HT, Applied Biosystems) and Lenti-X™ qRT-PCR Titration Kit (Clontech laboratories) and diluted to an appropriate concentartion. For the PCR sample preparation, lentivirus was incubated with DNase I (Lenti-X™ qRT-PCR Titration Kit components), and Lentiviral genomic RNA was purified and reverse-transcribed. Viral DNA was diluted to an appropriate concentration.

### Injection of viral vectors and fluorescent β-amyloid

5xFAD mice and control mice (C57BL/6 x SJL) were injected twenty days before imaging, EGFP- LC3 lentivirus (titer: 5.0 × 10^6^ vector genomes/ml, 5 μl) was injected into the retrosplenial dysgranular cortex (RSD, anteroposterior, −2.0 mm and mediolateral, 0.6 mm from bregma; depth, 1 mm), and the cerebellum cortex (anteroposterior, −7.0 mm and mediolateral, 1.2 mm from bregma; depth, 0.5 mm) of mice under anesthesia with 1.5% isoflurane. Human carboxytetramethylrhodamine (TAMRA)-β-amyloid 1–42, (100 μM, diluted by ACSF, DMSO, 0.1%; 1 μl) was injected into the RSD of mice under anesthesia with 1.5% isoflurane one day before imaging. Virus was delivered via a glass micropipette of 20–50 μm tip diameter made by capillary puller (P-1000 Pipette Puller, Sutter Instrument) under the conditions Heat at 741, pull power at 150, Velocity at 75, Delay at 1000 and Pressure at 400. 5 ul of viral solution was injected at the speed of 1 μl/min into RSD with an injection machine (FemtoJet^®^, eppendorf).

### *In vivo* imaging with two-photon microscopy

Two-photon imaging of autophagy was performed using a laser-scanning microscope system FV1000MPE2 (Olympus, Japan) equipped with an upright microscope (BX61WI, Olympus, Japan), a water-immersion objective lens (XLPlanN25xW; numerical aperture, 1.05), and a pulsed laser (MaiTaiHP DeepSee, Spectra Physics, USA). EGFP was excited at 890 nm and scanned at 500–550 nm. TAMRA-β-amyloid was excited at 1020 nm and scanned at 547–574 nm. The scanning area used for three-dimensional imaging was 100 × 100 μm (1 μm Z steps, 1,024 × 1,024 pixels, and digital zoom x 3). The LC3 labeled autophagosome in neuron of the mouse detected at cortical layer 1 were imaged through a thinned-skull window. To determine the function of autophagy, 1 μl of Aβ (TAMRA-β-amyloid 1-42: Cat;PMC-AK13-COS, 100 μM), was injected to same region as lentivirus EGFP-LC3. Images of EGFP-LC3-positive vesicles were analyzed for the number, size and signal intensity of autophagosome using IMARIS 7.2.2 (Bitplane, Switzerland).

### Fasting treatment of mice

Fasting treatment was performed for 0 to 48 hours during the imaging (from 12:00 am on day 1 to 12:00 am on day 3). Mice were allowed to drink water freely. During fasting treatment, body weight and blood glucose were measured with an weighing apparatus (A&D, EK-600i) and Blood Glucose Monitoring System (PILOT #10003001293).

### Immunohistochemistry

Mice were deeply narcotized with ether and fixed by perfusion of 4% paraformaldehyde. Brain tissues were sliced using a vibratome (HM 650V, Thermo) 30μm each. Immunohistochemistry was performed using antibodies (anti-NeuN, Millipore, MAB377; dilution, 1:200, anti-GFAP, Santa Cruz, SC-6170; 1:100, DAPI solution, Dojindo Laboratories, 340–07971, 1:10,000). To identify the character of EGFP-LC3 vesicles, immunohistochemistry was performed with autophagosome marker antibodies (anti-ATG7, Sigma-Aldlich, A2856, 1:200, anti-ATG12, Cell Signalling, 2011, 1:100).

For Aβ staining, sections were pretreated by boiling in 10 mM citrate buffer, pH 6.0 for 10 min, then incubated with proteinase K (100 g/ml) at 37 °C for 6 minutes or 0.5% Triton X-100 for 30 minutes. The sections were then blocked with 10% FBS in 50 mM Tris–HCl, pH 7.6, 150 mM NaCl (TBS) for 30 min. sectioned at 5 μm sections were stained with anti-Aβ antibody 82E1 (mouse, 1:1000, IBL) followed by incubation with biotin-labeled secondary antibody (1:500, Vector Laboratories), HRP-labeled avidin–biotin complex (Vector Laboratories), and the substrate 3,3' diaminobenzidine. The slides were counter-stained with hematoxylin and observed with a microscopy (Olympus BX53) and attached camera (Olympus DP72). For immunofluorescent staining the sections were stained with anti-Aβ 82E1 (mouse, 1:500, IBL), anti-LC3 (rabbit, 1:500, Sigma-Aldrich), or anti-LAMP2a (rabbit, 1:500, Abcam) overnight at 4 °C, then incubated with Alexa Fluor 488 labelled anti-mouse I gG (1:1000, Jackson ImmunoResearch) or Cy3 labeled anti-rabbit IgG (1:1000, Molecular Probes) for 1 h at room temperature. Images were obtained by confocal microscopy (Olympus FV1200 IX83).

### Positioning of image field

We used a stereomicroscope (Qlclick™, 74–0083-A0, Olympus) and a map of blood vessels to trace the same location of imaging area in RSD. First, we took images of specific area (Q-Capture Pro7, Olympus), and determine the location of EGFP-LC3 vesicles. The details are described in Figure legends (Supplementary Figure 3).

### Statistics

Two-way ANOVA followed by Tukey’s test was used for comparison of multiple groups at a similar time. The actual p-values are supplied in Supplementary Information (Supplementary Table 1).

### Ethics

All experimental procedures were performed in accordance with the protocols approved by the Committees on Human Ethics and Animal Experiments of Tokyo Medical and Dental University (2010-215C6, 0150270A, 0160328A).

## Additional Information

**How to cite this article**: Chen, X. *et al.* Fasting activates macroautophagy in neurons of Alzheimer's disease mouse model but is insufficient to degrade amyloid-beta. *Sci. Rep.* **5**, 12115; doi: 10.1038/srep12115 (2015).

## Supplementary Material

Supplementary Information
