# Supplementary material for: Fasting activates macroautophagy in neurons of Alzheimer’s disease mouse model but is insufficient to degrade amyloid-beta
Source: Sci Rep. 2015 Jul 14;5:12115. doi: 10.1038/srep12115 (PMC4648430; doi:10.1038/srep12115)
Supplement: Supplementary Information [file srep12115-s1.pdf]

Fasting activates macroautophagy in neurons of Alzheimer's disease mouse model but is insufficient to degrade amyloid-beta

Xigui Chen[1, $$], Kanoh Kondo[1, $$], Kazumi Motoki[1], Hidenori Homma[1] and Hitoshi Okazawa[1, 2, #]

1: Department of Neuropathology, Medical Research Institute, Tokyo Medical and Dental University, 1-5-45 Yushima, Bunkyo-ku, Tokyo 113-8510, Japan
2: Center for Brain Integration Research, Tokyo Medical and Dental University, 1-5-45 Yushima, Bunkyo-ku, Tokyo 113-8510, Japan

$$: co-first authors

#: Correspondence should be addressed to H.O.
E-mail: okazawa-tky@umin.ac.jp

Supplementary Information

Supplementary Figure 1

Expression of EGFP-LC3 in cerebellum

An image was reconstructed from three two-photon microscopy images of the cerebellar cortex of wild type mice (C57B6/J, 3 months of age) injected with EGFP-LC3 lentivirus to the cerebellum. EGFP-LC3 signals were extended along the orientation of Purkinje cell major dendrite. Granular cell layer (gra), Purkinje cell layer (PL), and molecular cell layer (mol) are indicated.

Supplementary Figure 2

Expression of EGFP-LC3 in Purkinje cells

EGFP-LC3 lentivirus was injected to cerebellar cortex of Ptf1a-Cre;Td-Tomato mice (3 months of age) to examine the EGFP-LC3 expression in Purkinje cells. Cerebellar tissues were fixed by perfusion of 4% paraformaldehyde, and fluorescence of EGFP and TdTomato was directly observed by two-photon microscopy. EGFP signals were observed both in cell body and dendrites of Purkinje cells. In addition, granule cells expressed EGFP-LC3.

Supplementary Figure 3

Changes of body weight and blood glucose during fasting

To confirm that fasting treatment was similarly loaded on 5xFAD and control mice, we checked body weight and blood glucose during fasting. The data showed the similar effects on two parameters by fasting. The numbers of mice are shown in the graphs. Mean +/- SE are shown. Two-way ANOVA followed by Tukey's test was used for comparison of multiple groups at a similar time. Asterisks (*) indicate the significant difference between fasting and non-fasting groups of the same genotype mice in the multiple comparisons ($p<0.05$).

Supplementary Figure 4

Circadian rhythm of autophagosome at the similar position in the brain

In chronological observation, we used vessels as hallmarks for positioning to

observe the same area in RSD. Representative images are shown.

Supplementary Figure 5

Quantitative analysis of autophagosome formation

Under the condition that water and food were supplied to mice ad libitum, autophagosomes labeled by EGFP-LC3 lentivirus infected before 2 weeks were chronologically observed. Four parameters were calculated and plotted in the graphs (mean +/- SE).

Supplementary Figure 6

Actual images used for the analyses in Figure 3A and B. Non-fasting wild type mice (upper left), fasting wild type mice (upper right), non-fasting 5xFAD mice (lower left), fasting 5xFAD mice (lower right).

Supplementary Table 1

Actual p-values in statistical tests are shown in the list.

Sup Fig 1

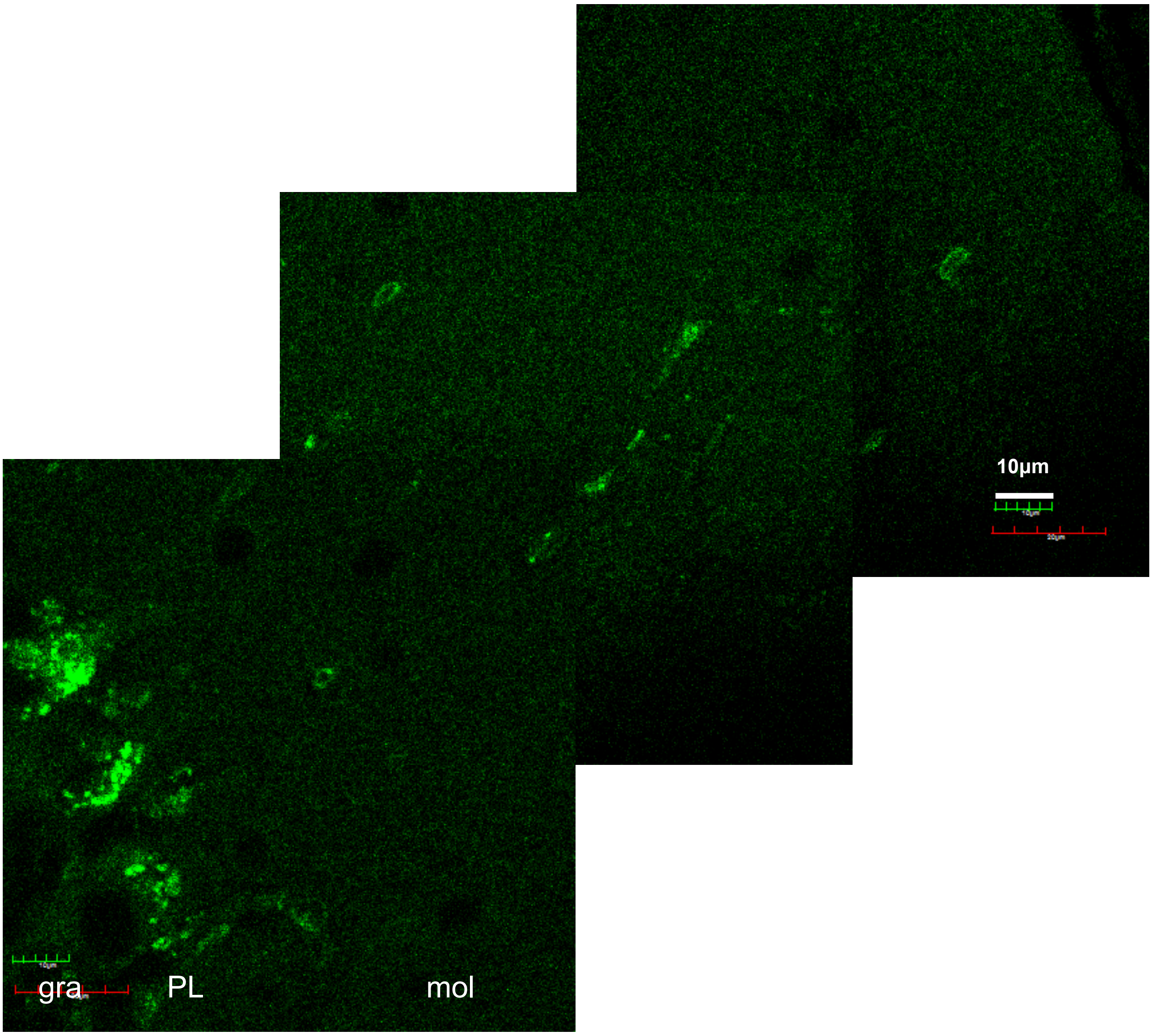

Sup Fig 2

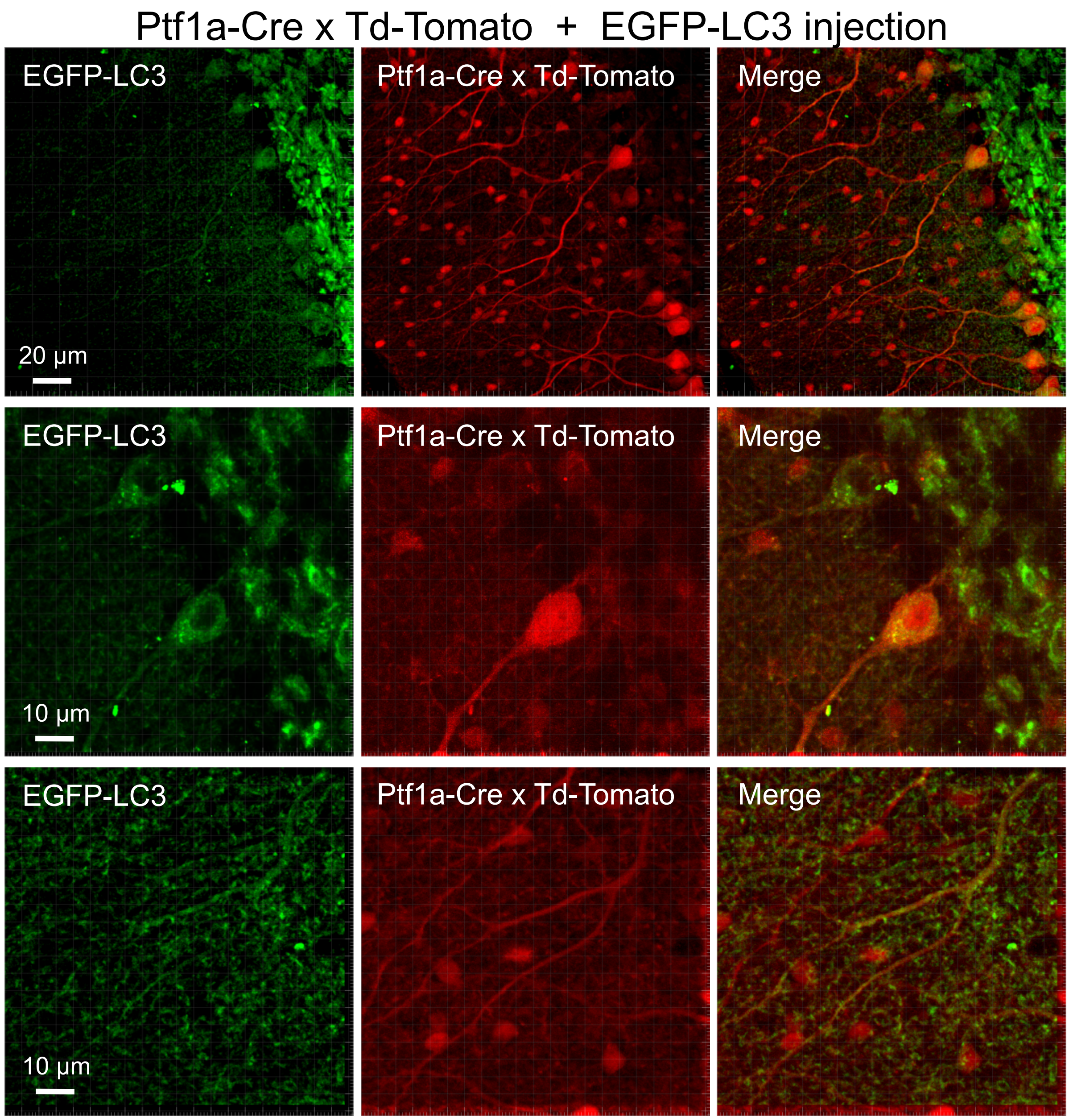

Ptf1a-Cre x Td-Tomato + EGFP-LC3 injection
EGFP-LC3
Ptf1a-Cre x Td-Tomato
Merge
20 µm
EGFP-LC3
Ptf1a-Cre x Td-Tomato
Merge
10 µm
EGFP-LC3
Ptf1a-Cre x Td-Tomato
Merge
10 µm

Sup Fig 3

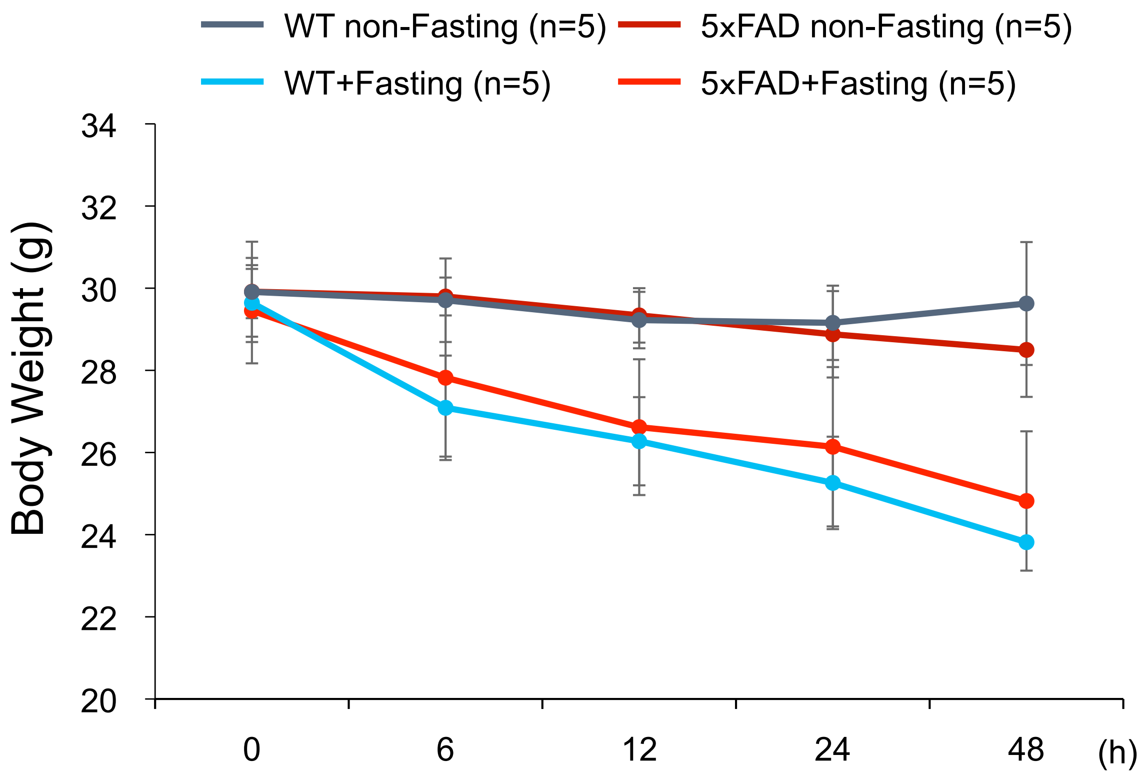

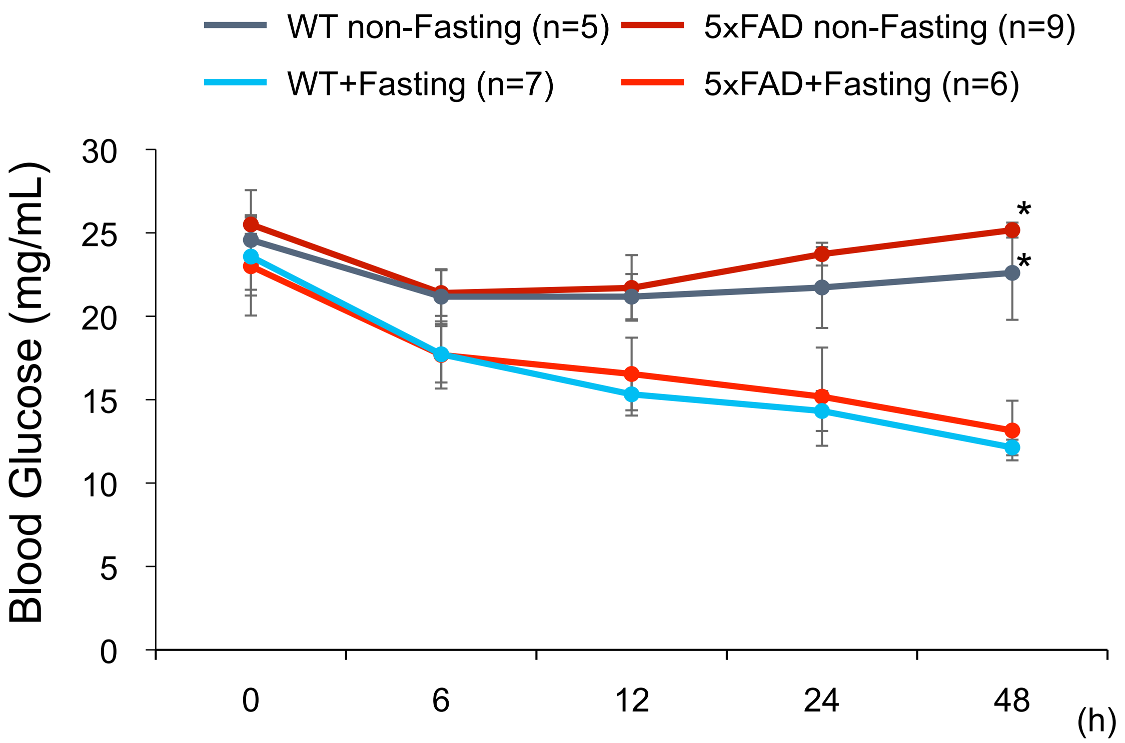

# or ## : WT+Fasting v.s. 5xFAD+Fasting
* or **: fasting + v.s. - in the same genotype
#, p<0.05; ##, p<0.01; *, p<0.05; **, p<0.01
by Two-way ANOVA followed by post hoc Tukey's test

Sup Fig 4

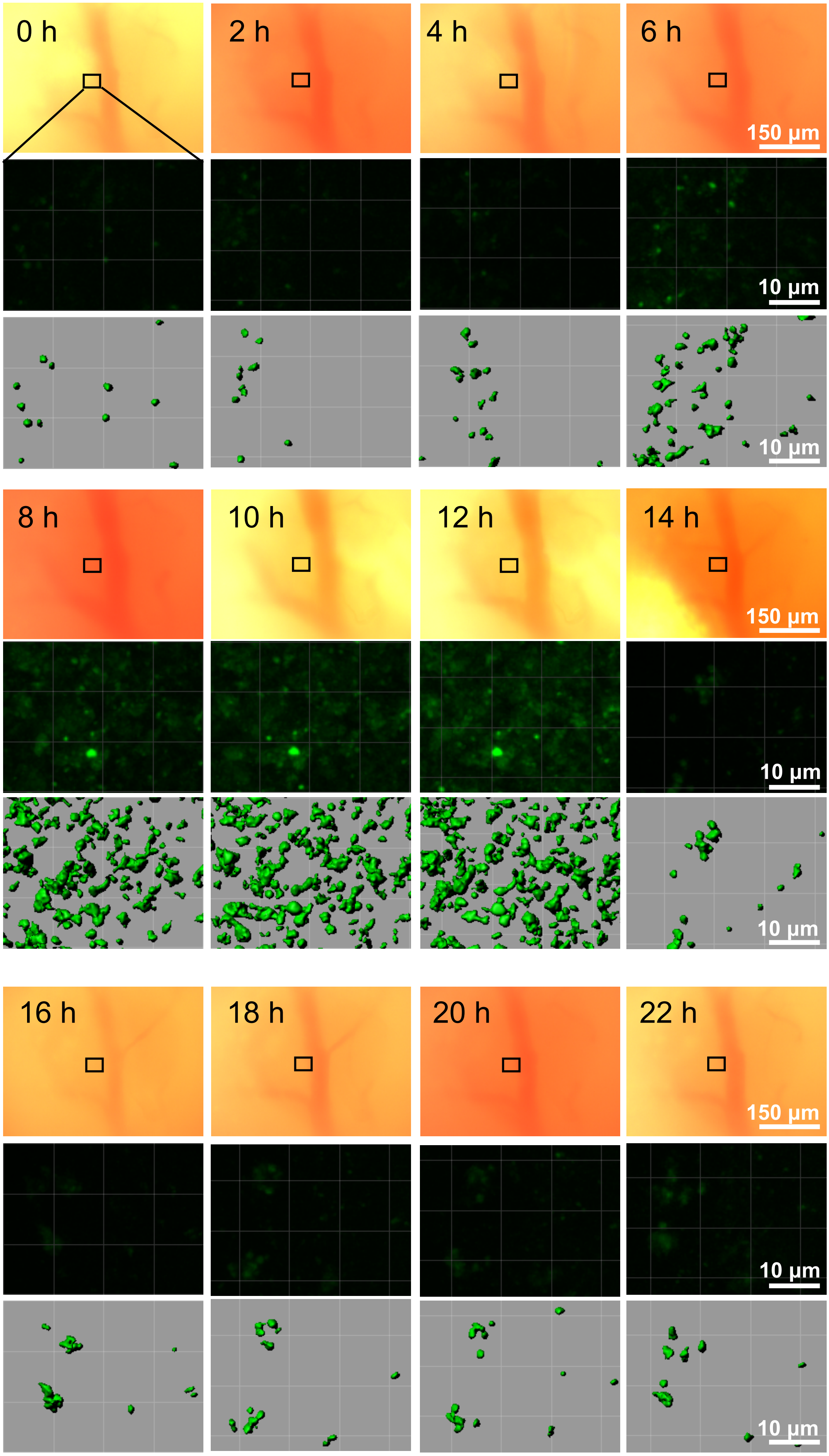

0 h
2 h
4 h
6 h
150 µm
10 µm
10 µm
8 h
10 h
12 h
14 h
150 µm
10 µm
10 µm
16 h
18 h
20 h
22 h
150 µm
10 µm
10 µm

Sup Fig 5

Circadian rhythm of autophagosome

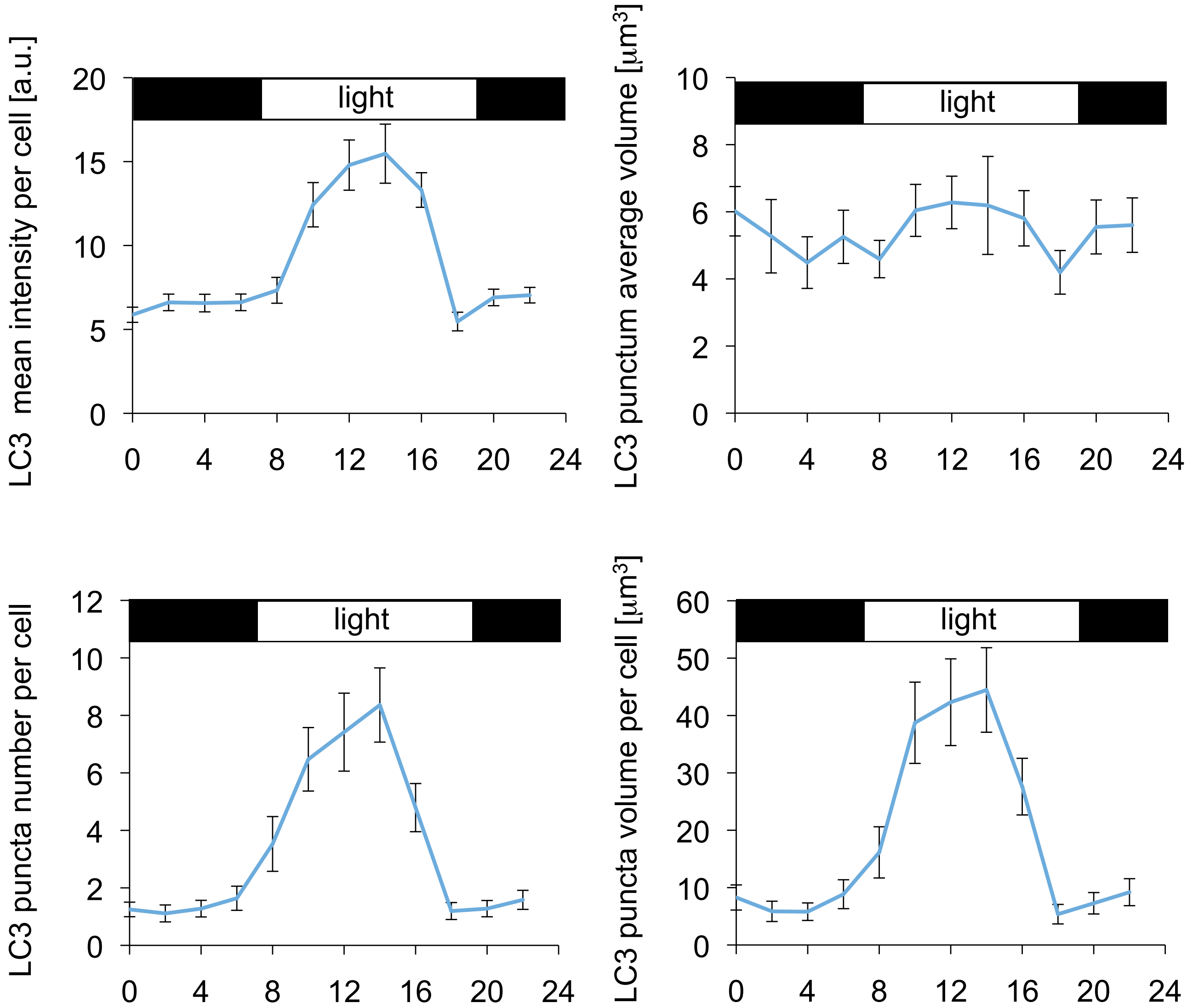

Mice, 3 months
Observed 3 weeks after lentiviral vector injection
n=34-37 cells

Sup Fig 6

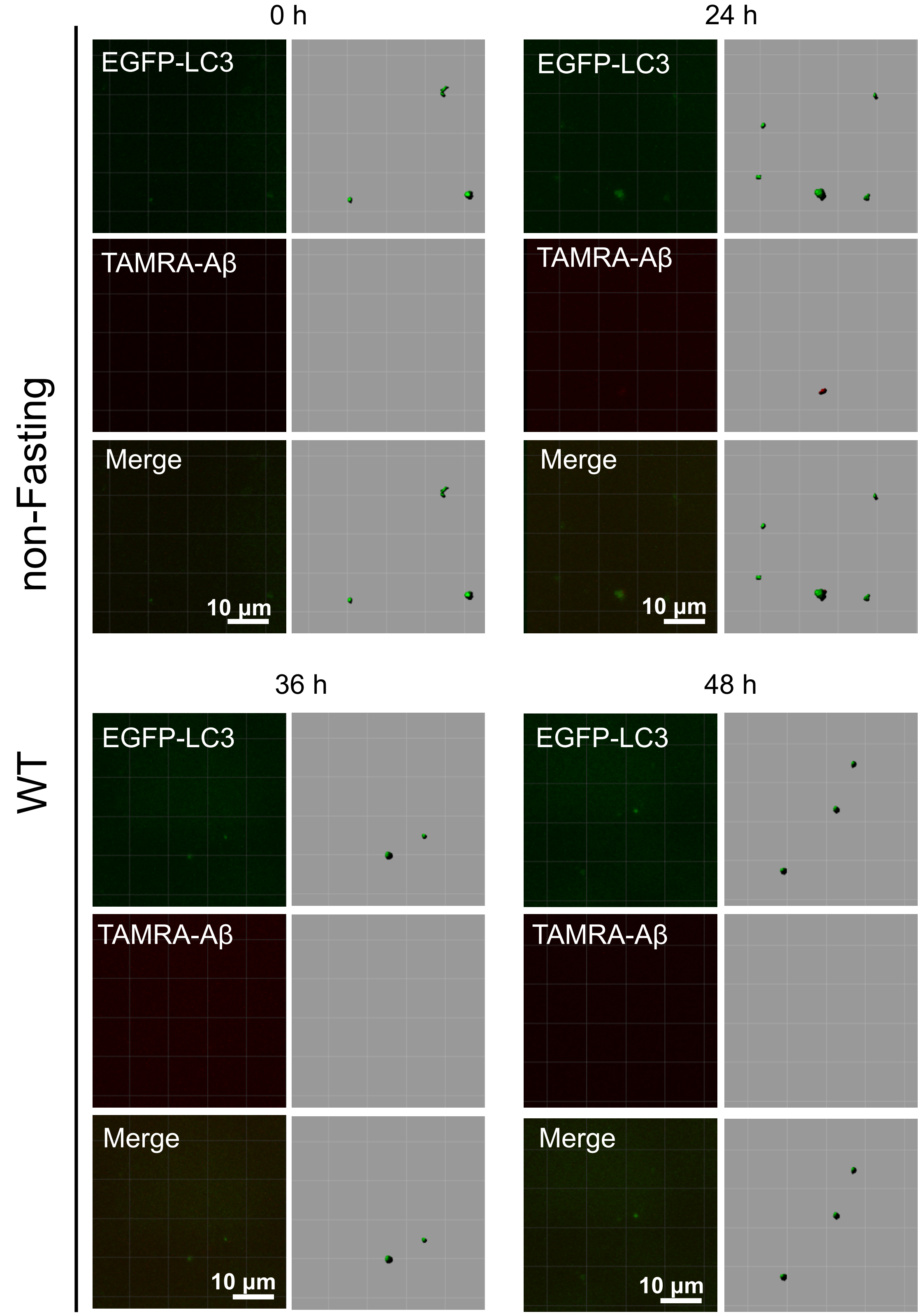

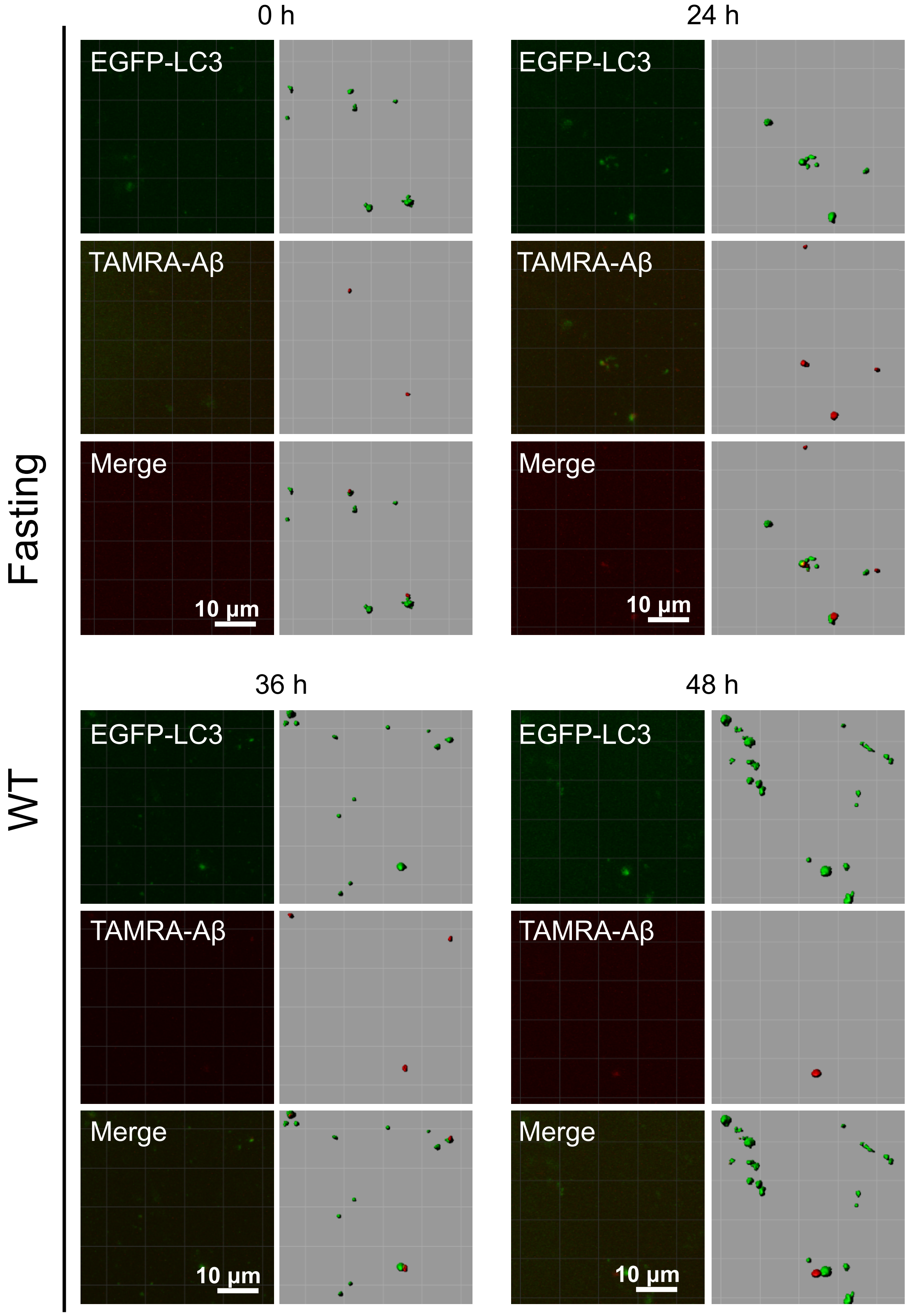
0 h
24 h
36 h
48 h
Fasting
WT
EGFP-LC3
TAMRA-Aβ
Merge
10 μm

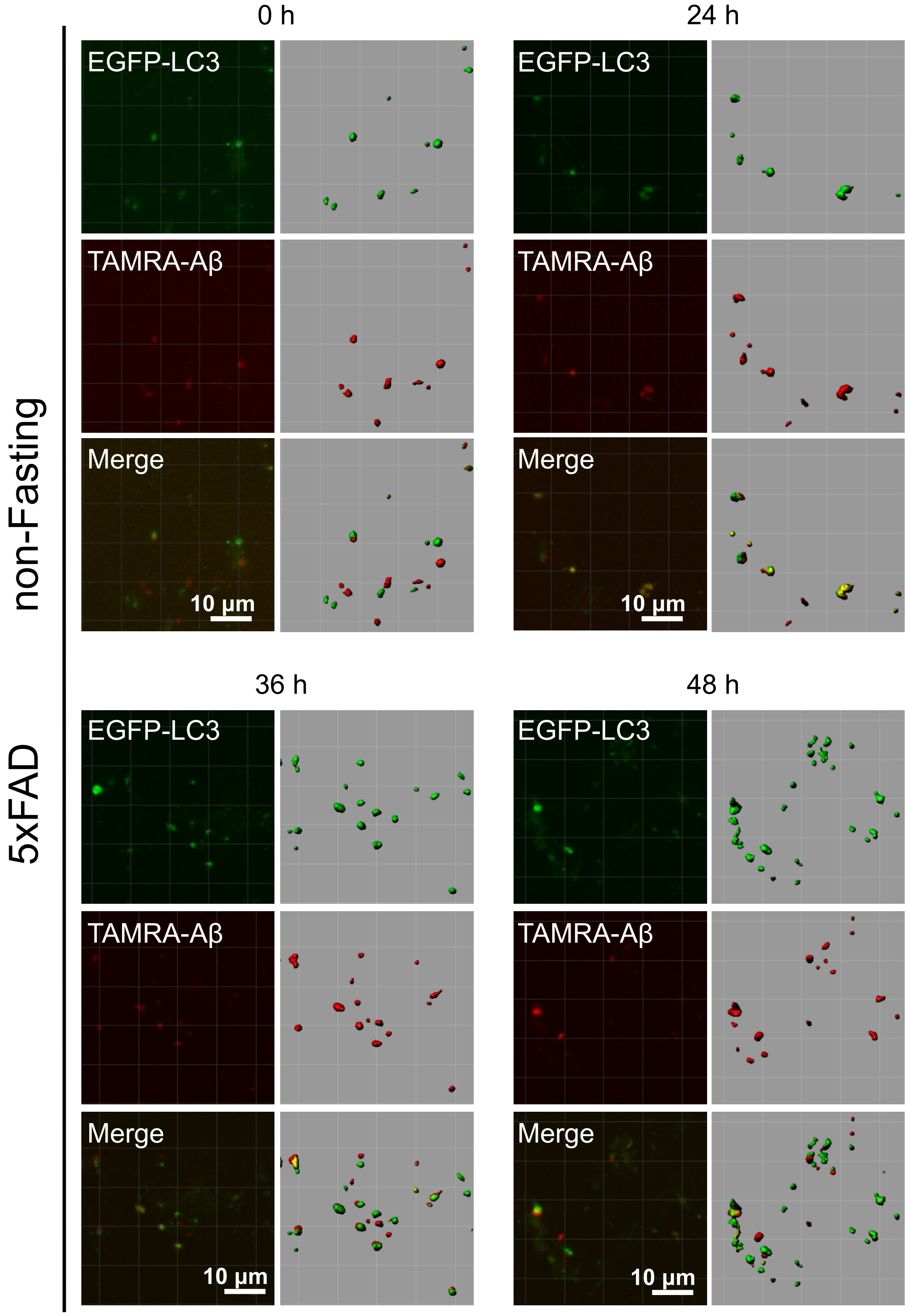

0 h
24 h
36 h
48 h
non-Fasting
5xFAD
EGFP-LC3
TAMRA-Aβ
Merge
10 μm

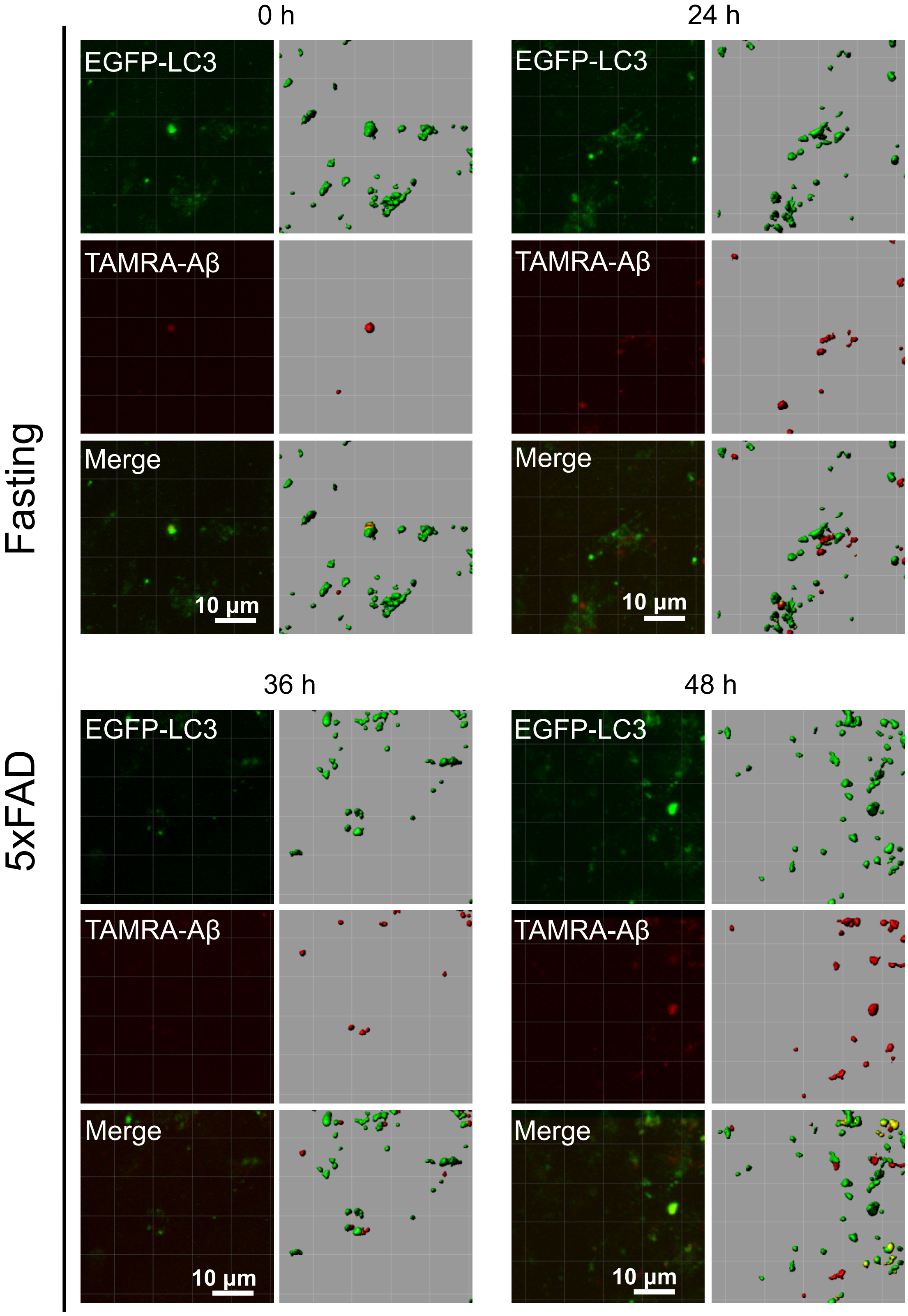
0 h
24 h
EGFP-LC3
TAMRA-Aβ
Merge
10 μm
Fasting
5xFAD
36 h
48 h
EGFP-LC3
TAMRA-Aβ
Merge
10 μm

Supplementary table 1: P-values in statistical tests.

F=Fasting, n=non-Fasting   p<0.05 (highlighted)

				P-value
Figure 2C: LC3 mean signal intensity	0h	2-way ANOVA	Genotype (G)	5.E-11
			Diet (D)	0.798
			Interaction (G × D)	0.869
		Tukey's test	5xFAD (n) vs 5xFAD (F)	0.991
			WT (F) vs 5xFAD (F)	5.E-06
			WT (n) vs 5xFAD (n)	2.E-05
			WT (n) vs WT (F)	0.999
	6h	2-way ANOVA	Genotype (G)	0.001
			Diet (D)	0.202
			Interaction (G × D)	0.050
		Tukey's test	5xFAD (n) vs 5xFAD (F)	0.970
			WT (F) vs 5xFAD (F)	0.717
			WT (n) vs 5xFAD (n)	0.001
			WT (n) vs WT (F)	0.100
	12h	2-way ANOVA	Genotype (G)	1.E-10
			Diet (D)	2.E-14
			Interaction (G × D)	0.004
		Tukey's test	5xFAD (n) vs 5xFAD (F)	9.E-13
			WT (F) vs 5xFAD (F)	2.E-10
			WT (n) vs 5xFAD (n)	0.030
			WT (n) vs WT (F)	0.001
	24h	2-way ANOVA	Genotype (G)	3.E-12
			Diet (D)	3.E-24
			Interaction (G × D)	1.E-05
		Tukey's test	5xFAD (n) vs 5xFAD (F)	7.E-15
			WT (F) vs 5xFAD (F)	4.E-14
			WT (n) vs 5xFAD (n)	0.043
			WT (n) vs WT (F)	1.E-05
	48h	2-way ANOVA	Genotype (G)	2.E-19
			Diet (D)	4.E-50
			Interaction (G × D)	1.E-12
		Tukey's test	5xFAD (n) vs 5xFAD (F)	0.E+00
			WT (F) vs 5xFAD (F)	0.E+00
			WT (n) vs 5xFAD (n)	0.247
			WT (n) vs WT (F)	0.E+00

				P-value
Figure 2C: LC3 puncta number per cell	0h	2-way ANOVA	Genotype (G)	0.019
			Diet (D)	0.860
			Interaction (G × D)	0.721
		Tukey's test	5xFAD (n) vs 5xFAD (F)	0.984
			WT (F) vs 5xFAD (F)	0.140
			WT (n) vs 5xFAD (n)	0.725
			WT (n) vs WT (F)	0.998
	6h	2-way ANOVA	Genotype (G)	9.E-05
			Diet (D)	0.445
			Interaction (G × D)	0.228
		Tukey's test	5xFAD (n) vs 5xFAD (F)	0.538
			WT (F) vs 5xFAD (F)	4.E-04
			WT (n) vs 5xFAD (n)	0.612
			WT (n) vs WT (F)	0.959
	12h	2-way ANOVA	Genotype (G)	6.E-06
			Diet (D)	0.006
			Interaction (G × D)	0.782
		Tukey's test	5xFAD (n) vs 5xFAD (F)	0.088
			WT (F) vs 5xFAD (F)	1.E-04
			WT (n) vs 5xFAD (n)	0.060
			WT (n) vs WT (F)	0.483
	24h	2-way ANOVA	Genotype (G)	0.289
			Diet (D)	4.E-06
			Interaction (G × D)	0.629
		Tukey's test	5xFAD (n) vs 5xFAD (F)	0.003
			WT (F) vs 5xFAD (F)	0.553
			WT (n) vs 5xFAD (n)	0.455
			WT (n) vs WT (F)	0.010
	48h	2-way ANOVA	Genotype (G)	0.040
			Diet (D)	3.E-12
			Interaction (G × D)	0.147
		Tukey's test	5xFAD (n) vs 5xFAD (F)	3.E-10
			WT (F) vs 5xFAD (F)	0.016
			WT (n) vs 5xFAD (n)	0.965
			WT (n) vs WT (F)	0.024

				P-value
Figure 2C: LC3 vesicle total volume per cell	0h	2-way ANOVA	Genotype (G)	0.023
			Diet (D)	0.725
			Interaction (G × D)	0.822
		Tukey's test	5xFAD (n) vs 5xFAD (F)	0.999
			WT (F) vs 5xFAD (F)	0.283
			WT (n) vs 5xFAD (n)	0.441
			WT (n) vs WT (F)	0.979
	6h	2-way ANOVA	Genotype (G)	0.065
			Diet (D)	0.981
			Interaction (G × D)	0.503
		Tukey's test	5xFAD (n) vs 5xFAD (F)	0.980
			WT (F) vs 5xFAD (F)	0.232
			WT (n) vs 5xFAD (n)	0.969
			WT (n) vs WT (F)	0.947
	12h	2-way ANOVA	Genotype (G)	0.003
			Diet (D)	0.419
			Interaction (G × D)	0.590
		Tukey's test	5xFAD (n) vs 5xFAD (F)	0.986
			WT (F) vs 5xFAD (F)	0.108
			WT (n) vs 5xFAD (n)	0.126
			WT (n) vs WT (F)	0.800
	24h	2-way ANOVA	Genotype (G)	0.008
			Diet (D)	0.037
			Interaction (G × D)	0.799
		Tukey's test	5xFAD (n) vs 5xFAD (F)	0.409
			WT (F) vs 5xFAD (F)	0.092
			WT (n) vs 5xFAD (n)	0.236
			WT (n) vs WT (F)	0.482
	48h	2-way ANOVA	Genotype (G)	0.021
			Diet (D)	6.E-06
			Interaction (G × D)	0.322
		Tukey's test	5xFAD (n) vs 5xFAD (F)	1.E-04
			WT (F) vs 5xFAD (F)	0.030
			WT (n) vs 5xFAD (n)	0.802
			WT (n) vs WT (F)	0.286

				P-value
Figure 3C: Red Volume per cell	0h	2-way ANOVA	Genotype (G)	1.E-07
			Diet (D)	0.894
			Interaction (G × D)	0.846
		Tukey's test	5xFAD (n) vs 5xFAD (F)	1.000
			WT (F) vs 5xFAD (F)	0.003
			WT (n) vs 5xFAD (n)	3.E-04
			WT (n) vs WT (F)	0.996
	24h	2-way ANOVA	Genotype (G)	2.E-09
			Diet (D)	0.256
			Interaction (G × D)	0.729
		Tukey's test	5xFAD (n) vs 5xFAD (F)	0.723
			WT (F) vs 5xFAD (F)	3.E-05
			WT (n) vs 5xFAD (n)	1.E-04
			WT (n) vs WT (F)	0.940
	36h	2-way ANOVA	Genotype (G)	2.E-12
			Diet (D)	0.059
			Interaction (G × D)	0.108
		Tukey's test	5xFAD (n) vs 5xFAD (F)	0.068
			WT (F) vs 5xFAD (F)	9.E-09
			WT (n) vs 5xFAD (n)	2.E-05
			WT (n) vs WT (F)	0.984
	48h	2-way ANOVA	Genotype (G)	1.E-20
			Diet (D)	2.E-07
			Interaction (G × D)	2.E-11
		Tukey's test	5xFAD (n) vs 5xFAD (F)	0.000
			WT (F) vs 5xFAD (F)	0.000
			WT (n) vs 5xFAD (n)	2.E-05
			WT (n) vs WT (F)	0.774

				P-value
Figure 3C: Yellow Volume per cell	0h	2-way ANOVA	Genotype (G)	0.004
			Diet (D)	0.908
			Interaction (G × D)	0.908
		Tukey's test	5xFAD (n) vs 5xFAD (F)	0.998
			WT (F) vs 5xFAD (F)	0.136
			WT (n) vs 5xFAD (n)	0.189
			WT (n) vs WT (F)	1
	24h	2-way ANOVA	Genotype (G)	5.E-04
			Diet (D)	0.001
			Interaction (G × D)	0.008
		Tukey's test	5xFAD (n) vs 5xFAD (F)	2.E-04
			WT (F) vs 5xFAD (F)	1.E-04
			WT (n) vs 5xFAD (n)	0.927
			WT (n) vs WT (F)	0.957
	36h	2-way ANOVA	Genotype (G)	0.004
			Diet (D)	0.025
			Interaction (G × D)	0.129
		Tukey's test	5xFAD (n) vs 5xFAD (F)	0.044
			WT (F) vs 5xFAD (F)	0.014
			WT (n) vs 5xFAD (n)	0.883
			WT (n) vs WT (F)	0.915
	48h	2-way ANOVA	Genotype (G)	0.014
			Diet (D)	5.E-03
			Interaction (G × D)	0.025
		Tukey's test	5xFAD (n) vs 5xFAD (F)	3.E-03
			WT (F) vs 5xFAD (F)	0.004
			WT (n) vs 5xFAD (n)	0.958
			WT (n) vs WT (F)	0.915

				P-value
Sup Fig 3C: Blood Glucose	0h	2-way ANOVA	Genotype (G)	0.277
			Diet (D)	0.649
			Interaction (G × D)	0.474
		Tukey's test	5xFAD (n) vs 5xFAD (F)	0.843
			WT (F) vs 5xFAD (F)	0.998
			WT (n) vs 5xFAD (n)	0.614
			WT (n) vs WT (F)	0.993
	6h	2-way ANOVA	Genotype (G)	0.839
			Diet (D)	0.104
			Interaction (G × D)	0.686
		Tukey's test	5xFAD (n) vs 5xFAD (F)	0.738
			WT (F) vs 5xFAD (F)	0.999
			WT (n) vs 5xFAD (n)	0.930
			WT (n) vs WT (F)	0.493
	12h	2-way ANOVA	Genotype (G)	0.742
			Diet (D)	0.015
			Interaction (G × D)	0.449
		Tukey's test	5xFAD (n) vs 5xFAD (F)	0.444
			WT (F) vs 5xFAD (F)	0.953
			WT (n) vs 5xFAD (n)	0.938
			WT (n) vs WT (F)	0.123
	24h	2-way ANOVA	Genotype (G)	0.937
			Diet (D)	0.020
			Interaction (G × D)	0.526
		Tukey's test	5xFAD (n) vs 5xFAD (F)	0.456
			WT (F) vs 5xFAD (F)	0.993
			WT (n) vs 5xFAD (n)	0.914
			WT (n) vs WT (F)	0.170
	48h	2-way ANOVA	Genotype (G)	0.770
			Diet (D)	4.E-04
			Interaction (G × D)	0.700
		Tukey's test	5xFAD (n) vs 5xFAD (F)	0.019
			WT (F) vs 5xFAD (F)	0.991
			WT (n) vs 5xFAD (n)	0.994
			WT (n) vs WT (F)	0.043

				P-value
Sup Fig 3C: Body Weight	0h	2-way ANOVA	Genotype (G)	0.980
			Diet (D)	0.739
			Interaction (G × D)	0.928
		Tukey's test	5xFAD (n) vs 5xFAD (F)	0.990
			WT (F) vs 5xFAD (F)	0.999
			WT (n) vs 5xFAD (n)	1.000
			WT (n) vs WT (F)	0.998
	6h	2-way ANOVA	Genotype (G)	0.522
			Diet (D)	0.116
			Interaction (G × D)	0.818
		Tukey's test	5xFAD (n) vs 5xFAD (F)	0.748
			WT (F) vs 5xFAD (F)	0.979
			WT (n) vs 5xFAD (n)	1.000
			WT (n) vs WT (F)	0.527
	12h	2-way ANOVA	Genotype (G)	0.504
			Diet (D)	0.033
			Interaction (G × D)	0.924
		Tukey's test	5xFAD (n) vs 5xFAD (F)	0.413
			WT (F) vs 5xFAD (F)	0.996
			WT (n) vs 5xFAD (n)	1.000
			WT (n) vs WT (F)	0.313
	24h	2-way ANOVA	Genotype (G)	0.483
			Diet (D)	0.034
			Interaction (G × D)	0.683
		Tukey's test	5xFAD (n) vs 5xFAD (F)	0.541
			WT (F) vs 5xFAD (F)	0.966
			WT (n) vs 5xFAD (n)	0.999
			WT (n) vs WT (F)	0.232
	48h	2-way ANOVA	Genotype (G)	0.890
			Diet (D)	0.009
			Interaction (G × D)	0.471
		Tukey's test	5xFAD (n) vs 5xFAD (F)	0.293
			WT (F) vs 5xFAD (F)	0.961
			WT (n) vs 5xFAD (n)	0.935
			WT (n) vs WT (F)	0.080
